# Effect of traditional processing methods on the physicochemical, nutritional, and functional properties of little millet (*Panicum sumatrense* L.) for functional food applications

**DOI:** 10.3389/fnut.2026.1840125

**Published:** 2026-06-23

**Authors:** Chaitra Chidambar Kulkarni, Jayaramegowda Thejaswi, Bharat Kumar, Seetur Radhakrishna Pradeep, Susmita Barman

**Affiliations:** 1 Department of Biotechnology and Crop Improvement, KRCCH, University of Horticultural Sciences, Bagalkot, Karnataka, India; 2 Division of Yoga and Life Sciences, Swami Vivekananda Yoga Anusandhana Samsthana (S-VYASA), Bangalore, Karnataka, India; 3 Department of Biochemistry, CSIR- Central Food Technological Research Institute, Mysuru, Karnataka, India; 4 Department of Obstetrics and Gynecology, University of Nebraska Medical Center, Omaha, NE, United States

**Keywords:** carbohydrate digestibility, functional foods, glycemic index, little millet, pasting profile

## Abstract

**Introduction:**

Little millet (*Panicum sumatrense* L.) is an underutilized, climate-resilient cereal with high nutritional potential; however, information on how conventional processing methods modify its physicochemical, nutritional, and functional properties for food applications is limited.

**Methods:**

This study aimed to evaluate the effects of germination (48 h), steaming (0 and 1.5 kscg for 15 min), and roasting (165 ± 2 °C for 75 s) on the physical, nutritional, and functional characteristics of little millet.

**Results:**

Processing methods markedly altered grain structure, density, and hardness of millet grains. Germination reduced bulk density (by 36.3%) and hardness while improving *in vitro* carbohydrate digestibility (79.98% at 120 min compared to 75.40% in raw millet). In contrast, steaming and roasting increased hardness (up to 5.32 kg) and enhanced nutritional quality of the grain, including protein (up to 32.3%), dietary fiber (up to 44.9%), phosphorus (up to 32.4%), and oryzanol (up to 35.4%) content, with a concomitant reduction in carbohydrate and amylose content. Functional properties of the processed grains were also modified. Heat-treated methods show lower peak and setback viscosities, indicating improved processing stability in the grain. Notably, carbohydrate digestibility decreased with roasting (64.81% at 120 min) and steam processing in the samples, suggesting potentiality of the processing methods for low-glycaemic applications.

**Discussion:**

Unlike previous studies that focused on limited processing effects, this study addresses the need to enhance the underutilized millets through simple, scalable processing techniques and provides a comprehensive evaluation linking compositional and functional changes to end-use applications. Germinated millet is suitable for easily digestible formulations such as weaning foods, whereas roasted and steam-processed millet flours are promising for developing low-glycaemic, high-fibre functional foods targeting metabolic disorders. Overall our findings spotlight the prospect of these conventional processing methods by enhancing the nutritional and functional value of little millet, supporting its wider utilization in health-promoting food products and their impact on our healthy lifestyle.

## Highlights

- Effect of traditional processing methods such as germination, steaming and roasting on the little millet physicochemical, nutritional, and functional properties were investigated.
- Processing methods profoundly alter the physiochemical and functional properties of little millet.
- Germination reduced bulk density and hardness; steaming and roasting increased porosity and hardness of grains.
- Steaming and roasting methods significantly reduced amylose content, carbohydrate digestibility, and peak viscosity of millet.

## 1 Introduction

Millets are climate-resistant minor cereals and form the staple food for a large segment of the population, mainly those with low socio-economic status, in India, Africa, Asia and other countries. They are considered crops for food security because of their sustainability in adverse environmental conditions such as saline soils, drought, and high-temperature conditions. Nutritionally, *Panicum sumatrense* stands out for its exceptional composition, being rich in carbohydrates (70–80%), balanced protein (9–14%), and high dietary fibre (9–16%). It is a starchy food with a 25:75 amylose to amylopectin ratio and is a fairly good source of lipids (3–6%), with about 50% of the lipids present as polyunsaturated fatty acids (1). Thus, it is positioned as a promising candidate for functional food development with potential relevance for non-communicable diseases such as diabetes and obesity (2).

Little millet (*Panicum sumatrense*) is an important minor millet grown in low and moderate rainfall areas of India, Pakistan, Nepal, Southeast Asia, Central China, and other subtropical regions (3). Insect pests do not readily attack little millet grains during prolonged storage due to the presence of proteinaceous defence factors in the grain endosperm (4). Its tolerance to harsh environmental conditions, high nutritional value, low cost, and good storage stability make it a potential crop for ensuring food security.

The physical properties of millet, like other grains and seeds, are essential for designing equipment for harvesting, processing, and storage. These properties influence handling characteristics, heat and mass transfer, and processing efficiency. The nutritional and functional qualities of little millet have been reported in earlier studies (5, 6). However, its utilization remains largely confined to traditional and tribal populations due to limited processing technologies and lack of scientific information, which restricts its wider commercialization. Moreover, information on how traditional processing alters these properties in little millet remains limited.

Recent studies have emphasized the importance of advanced and optimized processing techniques to improve grain functionality. For instance, ultrasound-assisted hydration has been shown to significantly enhance water absorption, germination, and antioxidant capacity in oat seeds (7). Similarly, emerging treatments such as cold plasma and UV irradiation improve sprout growth and nutritional quality in alfalfa sprouts (8), while natural pre-treatments like limonene enhance safety and prebiotic functionality of fresh amaranth sprouts (9). In addition, roasting has been reported to significantly influence biochemical traits, volatile compounds, and sensory attributes through structural transformations in pistachio nuts (10).

However, information on the effect of processing method on little millet is still limited. The effect of processing on the nutraceutical and antioxidant properties of little millet extracts has been previously reported (5). In the present study, we focus on the changes in physical, nutritional, and functional properties of little millet during processing, with emphasis on improving digestibility and potentially lowering the glycaemic response. This study may support its utilization as a sustainable, gluten-free, and high-fibre ingredient for food industry applications.

## 2 Materials and methods

### 2.1 Materials

Little millet (CO-Samai-4 variety) used in the present study was procured from Tamil Nadu Agricultural University, Coimbatore, India. Initial care was taken to procure grains from a single batch. Grains were cleaned to remove extraneous matter, shriveled, damaged grains, and stored in cool and dry place until use. All chemicals and reagents were purchased from Sigma-Aldrich Chemical Co. (St. Louis, MO, USA) or SISCO Research Laboratories (Mumbai, India) and were of analytical grade and highest purity.

### 2.2 Methods

#### 2.2.1 Processing of millet grains

Millet grains were soaked for ~16 h in water in the ratio of 1:3 (w/v) at an ambient temperature of 30 °C. Grains so treated were then separated into four lots for further processing as mentioned in our previous study (5).

i) Germination

Soaked grains were placed on wet muslin cloth and allowed to germinate at 25 ± 2 °C in BOD incubator (Model#BOD-200, M.S. Scientific Instruments Work, New Delhi, India) for 48 h, with regular wetting (5).

ii) Steaming

Soaked grains were subjected to steaming in an autoclave (Model # PSM A4, PSM Scientific Instruments Pvt. Ltd., Bengaluru, India) under 0 kilogram-force per square centimetre gauge (kscg) and 1.5 kilogram-force per square centimetre gauge (kscg) for 15 min for 15 min (5).

iii) Roasting

Soaked grains were roasted in sand with the ratio of (3:1) in electrically heated bowl roaster (Brand: Janatha Roaster, Sri Venkateshwara Industry, Mysuru, Karnataka, India) at 165 ± 2 °C for 75 s (5).

Thereafter, the immediate moisture content of the processed and raw (unprocessed) millet grains was determined. Bulk batches of raw and processed grains were dried in a mechanical try drier (Catalogue# TD-12-1, MRS Scientific Instruments, Soorapattu, Villupuram, Tamil Nadu, India) at 40 ± 2 °C for 3 h, decorticated using a laboratory grain polisher (Model: TM-05C, Satake Corporation, Japan), and pulverized in a communition mill (Universal Engineering Works, Mysuru, India) separately to obtain some flour to pass through a 250 μm size aperture sieve and stored at −20 °C in polythene pouches until further analysis as mentioned in our previous study (5).

### 2.3 Physical properties

#### 2.3.1 Immediate moisture content

Moisture content was measured as per the AACC Method (11) after treatment, immediately before drying and expressed as immediate moisture content (%).

#### 2.3.2 Equilibrium moisture content upon soaking (EMC-S)

Raw and processed millet grains were soaked in distilled water at ambient temperature (~30) for 30 min, drained off water, the grain surface was wiped with tissue paper, and the moisture content was measured according to the AACC method (11).

#### 2.3.3 Geometric mean diameter and sphericity

Geometric mean diameter (Dg) and Sphericity (Ø) of raw and processed millet were measured in millimeter (mm) and percentage (%) and calculated by using the following relationship (12).


$$\mathrm{Dg}={\left(\mathrm{LWT}\right)}^{1/3}$$

$$Ø={\left(\mathrm{LWT}\right)}^{1/3}/L$$

Were, L: Length (mm); W: Width (mm); T: Thickness (mm).

#### 2.3.4 Bulk density

Bulk density of raw and processed millet grains was calculated by determining the ratio of the mass of the grains to its bulk volume. It was determined by filling a 1,000 mL container with grains dropped from a height of about 15 cm, skimming off the top level, then weighing the contents and expressed in terms of kilogram per cubic meter (kg/m^3^). The average of at least three measurements was recorded.

#### 2.3.5 True density

The true density defined as the ratio of the mass of the grains to its grain volume, was determined for raw and processed millet using the liquid displacement method. 50 mL of kerosene was placed in a 100 mL graduated measuring cylinder and millet grains (5 g) were immersed. Kerosene was preferred over water due to its lower specific gravity and non-absorbent nature. The amount of displaced kerosene was recorded from the graduated scale of the cylinder. The ratio of the weight of grains to the volume of displaced kerosene gave the true density and expressed in terms of kg per cubic meter (kg/m^3^).

#### 2.3.6 Porosity

Porosity, the fraction of the space in bulk grains not occupied by the grains, was calculated from the values of true density and bulk density using following relationship (12).


$$\varepsilon =(1–\rho_{b})/\rho_{t}\times 100$$

Were, ε: Porosity (%); ρ_b_: Bulk density (kg/m^3^); ρ_t_: True density (kg/m^3^).

#### 2.3.7 Hardness

Hardness of grain was determined using a manually operated Kiya grain hardness tester (Model # 174886, Kiya Seisakusho Ltd., Tokyo, Japan) by crushing 30 individual grains. Hardness was reported by eliminating the extreme and odd values and then calculating the mean of the remaining readings.

#### 2.3.8 Scanning electron microscopy (SEM)

A scanning electron micrograph was obtained at 20 kV using a scanning electron microscope (Model# LEO 435 VP, LEO Electron Microscopy Ltd., Cambridge, UK). A double-sided conducting adhesive tape pasted onto a metallic stub was used to mount the grain samples. A thin section of grains was gold coated (~100 A°) with a sputter coater (Model#5001, Polaron Sputter Coat System, England) and scanned under different magnifications to get a clear pattern and used for comparison.

### 2.4 Nutrient composition

Raw and processed millet flours were analyzed for moisture, protein, fat, and total ash content by AACC method (11), dietary fibers (13) and carbohydrate by different methods. The energy value (kcal/100 g) was determined using a formula: (4 × protein) + (9 × fat) + (4 × carbohydrate).

#### 2.4.1 Total and soluble amylose content

Total and soluble amylose contents were determined according to the method described in reference (14).

#### 2.4.2 Total phosphorus content

Total phosphate released is measured using a modified colorimetric method (15) and given as milligrams of phosphorus per 100 g of sample material (mg/100 g).

#### 2.4.3 Oryzanol content

Oryzanol content was determined spectrophotometrically in the petroleum ether (60–80 °C) extract of millet flour by scanning the wavelength range of 220–420 nm. The maximum absorbance was recorded at 314 nm using spectrophotometer (Model Shimadzu UV-1800, Shimadzu Corporation, Kyoto, Japan) and the oryzanol content was calculated based on the absorbance of a 1% standard oryzanol solution at 314 nm (16).

### 2.5 Functional properties

#### 2.5.1 Gel consistency

Gel consistency of millet flour was determined according to the method described in (17) and expressed in terms of centimetre (cm).

#### 2.5.2 Sediment volume

Sediment volume was determined according to the method described in (18) and expressed in terms of percentage (%).

#### 2.5.3 Swelling and solubility

The swelling power and solubility pattern of raw and processed millet flour were determined as per the method (19) and expressed in terms of gram percentage (g%).

#### 2.5.4 Pasting profile

A 12% (w/v) slurry of each of the composite flour taken in the bowl of the amylograph was heated to raise temperature from 30 to 92 °C at the rate of 7.5 °C/min, maintained at 92 °C for 1 min and cooled to 50 °C at the same rate and the changes in the viscosity was recorded in a Brabender Micro Visco-amylograph (Model #803202, Brabender, Duisburg, Germany).

#### 2.5.5 *In vitro* carbohydrate digestibility

Carbohydrate digestibility of the defatted samples (100 mg) was determined by enzymatic digestion by treating with termamyl, pepsin, pancreatin and amyloglucosidase sequentially for different intervals of time, according to the method (20). Glucose released was estimated by using dinitrosalycilic acid method.

#### 2.5.6 Colour measurement

Colour value of flour samples was determined using Hunter colour measuring system L*, a*, b* scales (Lab scan XE model, M/s Hunter associate laboratory Inc., Reston-V. A., USA) with a view angle of 2°. In the Hunter system L* indicates brightness or whiteness, positive a* value indicates redness and negative value indicates greenness. Positive b* value indicates yellowness and negative b* indicates blueness, ∆E indicates the overall average colour.

### 2.6 Data analysis

All analyses were performed in triplicate, and the results were expressed as mean values ± standard deviation using GraphPad Instate statistical software (GraphPad Software Inc., La Jolla, Calif., USA). Statistical significance was determined by one-way analysis of variance (ANOVA) using Dunnett test. Values with *p* < 0.05 were considered statistically significant.

## 3 Results

### 3.1 Physical properties of raw and processed millet grains

The physical properties of raw and processed millet grains are presented in Table 1. Processing treatments significantly affected the moisture content of millet grains. Immediate moisture content increased by 3.38-fold after germination, 3.16- and 3.20-folds after steam processing treatments, and 1.79-fold after roast processing compared to the raw grains.

**Table 1:** Effect of processing on physical properties of little millet (whole grain).

Parameters	Raw	Germinated	Steamed (0 kscg)	Steamed (1.5 kscg)	Roasted
Immediate moisture content (%)	10.22 ± 0.65^a^	34.59 ± 0.90^b^	32.38 ± 0.55^c^	32.75 ± 0.50^d^	18.37 ± 0.32^e^
EMC-S (%)	27.39 ± 1.66^a^	32.01 ± 0.90^b^	30.15 ± 0.56^a^	39.27 ± 0.90^c^	32.68 ± 1.53^d^
Geometric mean diameter (mm)	1.54 ± 0.01^a^	1.69 ± 0.10^a^	1.60 ± 0.04^a^	1.63 ± 0.01^a^	1.57 ± 0.15^a^
Sphericity index (%)	0.640 ± 0.01^a^	0.701 ± 0.10^a^	0.661 ± 0.01^a^	0.651 ± 0.02^a^	0.683 ± 0.01^a^
Thousand grain mass (g)	2.60 ± 0.25^a^	2.42 ± 0.18^a^	2.49 ± 0.22^a^	2.43 ± 0.05^a^	2.46 ± 0.10^a^
Bulk density (kg/m^3^)	734.5 ± 6.53^a^	468.0 ± 6.15^b^	643.5 ± 3.50^c^	543.1 ± 6.98^d^	640.0 ± 6.17^e^
True density (kg/m^3^)	1.11 ± 0.16^a^	1.17 ± 0.04^a^	1.23 ± 0.10^a^	1.29 ± 0.17^a^	1.21 ± 0.03^a^
Porosity (%)	33.78 ± 0.50^a^	51.75 ± 1.16^b^	46.39 ± 1.20^c^	56.55 ± 0.42^d^	36.00 ± 0.75^a^
Hardness (kg)	1.51 ± 0.15^a^	0.76 ± 0.07^a^	2.64 ± 0.35^b^	5.32 ± 0.20^c^	3.22 ± 0.19^d^

Values are mean ± standard deviation of three independent determinations. Values in the same row (in each parameter) with different superscripts (a, b, c, d, e) are significantly different at *p* < 0.05 when raw compared with the respective process. %, percentage; EMC-S, equilibrium moisture content upon soaking; g, gram; kg, kilogram; kscg, kilogram-force per square centimetre gauge; m^3^, cubic meter; mm, millimetre.

Equilibrium moisture content at saturation (EMC-S) showed notable increases across all processing methods. Germination resulted in a 16.9% increase, while steaming showed increases of 10.1 and 43.4%. Roasting treatment increased EMC-S by 19.3% compared to raw grains.

The geometric mean diameter and sphericity index showed no significant differences between processed and raw grains, indicating that they maintained their circular nature. The 1,000-grain weight and true density decreased during germination and heat processing treatments, though these changes were not statistically significant.

Bulk density of raw millet was 734.5 kg/m^3^, which decreased during all processing treatments: germination (36.3% reduction), steam treatment (12.4, and 26.0% reduction), and roasting (12.9% reduction).

Porosity increased during all processing methods, with the highest increase observed in 1.5 kscg steam processed millet grains (67.4% compared to raw millet). Further, processing methods significantly affected grain hardness, with values ranging from 0.76 to 5.32 kg. Germination caused a significant reduction in hardness from 1.51 kg (raw) to 0.76 kg. Conversely, steaming and roasting significantly increased the hardness to 2.64–5.32 kg in respective treatment grains.

**Figure 1:**
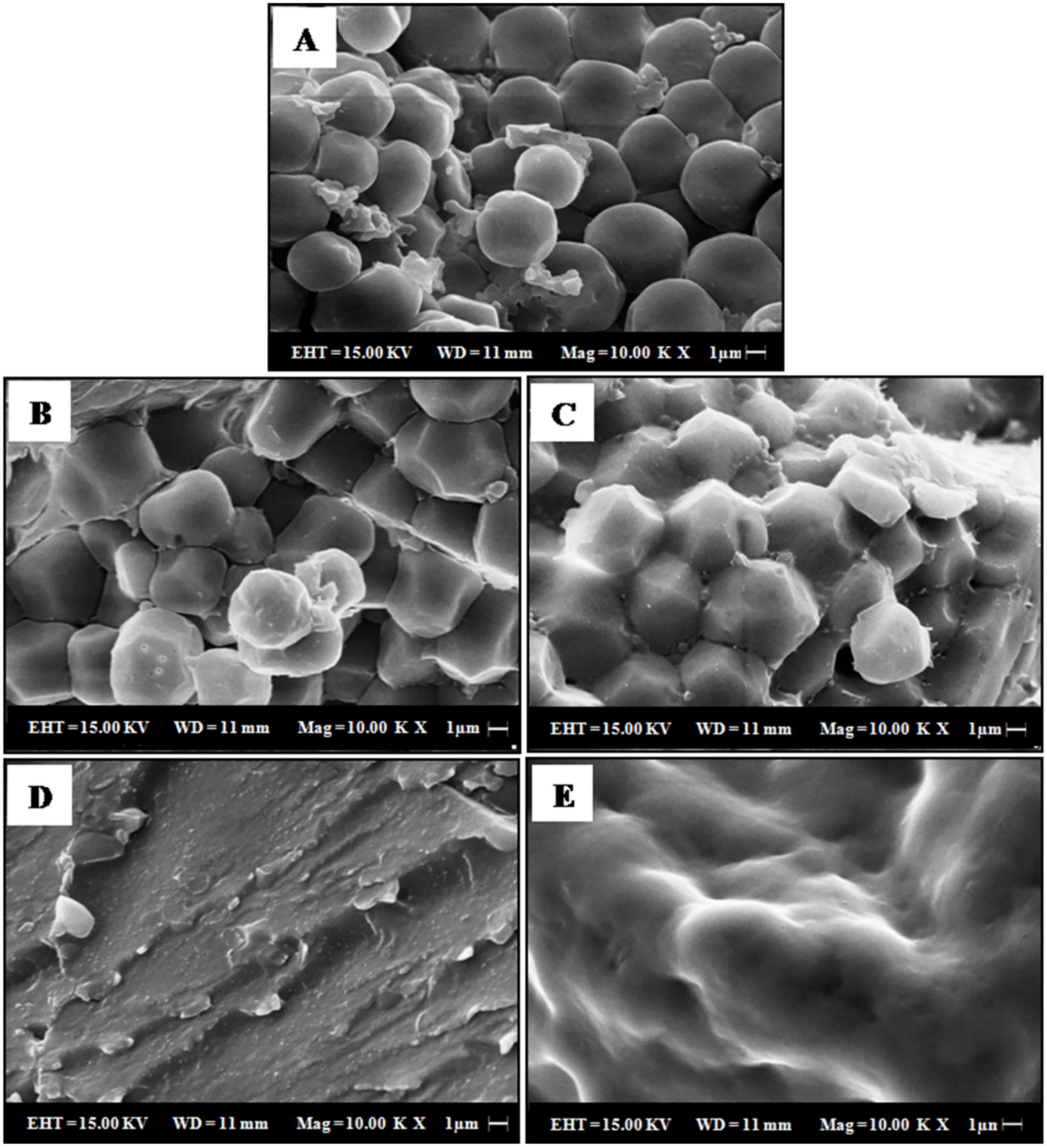
Scanning electron microscopic (SEM) picture of raw and processed little millet (10.00 K×). **(A)** Raw, **(B)** germinated, **(C)** steamed (0 kscg), **(D)** steamed (1.5 kscg), **(E)** roasted. Representative picture of three independent experiments. kscg, kilogram-force per square centimetre gauge.

Scanning electron microscopy (SEM) revealed noticeable structural variations among the different millet treatments. Raw millet showed, intact, well-defined spherical granules with a compact and uniform arrangement (Figure 1A). Whereas germination of grains caused granule expansion due to high moisture absorption, producing more compact polygonal shapes (Figure 1B). Mild steaming (0 kscg) preserved polyhedral granules with minimal change (Figure 1C). In contrast, harsh steaming (1.5 kscg) resulted in extensive gelatinization, swelling, and deformation of starch granules, leading to the formation of fused and interconnected structures (Figure 1D). Roasting resulted in complete gelatinization and retrogradation of amylose, forming crystalline structures (Figure 1E).

### 3.2 Nutritional composition

The nutritional composition of raw and processed millet grains is presented in Table 2. Protein content in raw and processed millet ranged from 10.04 to 13.28%. Processing of millet grains showed significantly (*p* < 0.05) increase in protein content compared to raw, with the maximum increase in roast processed millet grain (32.3%). Both steam-processed flour, 0 kscg (20.0%) and 1.5 kscg (30.0%), also showed substantial increase in protein content compared to raw millet.

**Table 2:** Effect of processing on nutrient composition of little millet.

Parameters	Raw	Germinated	Steamed (0 kscg)	Steamed (1.5 kscg)	Roasted
Moisture (g %)	10.22 ± 0.60^a^	10.13 ± 0.91^a^	9.24 ± 0.80^a^	8.34 ± 0.15^a^	8.05 ± 0.75^a^
*Protein (g %)	10.04 ± 0.10^a^	12.72 ± 0.22^b^	12.05 ± 0.35^c^	13.05 ± 0.43^d^	13.28 ± 0.50^e^
Fat (g %)	5.29 ± 0.22^a^	5.56 ± 0.25^a^	5.19 ± 0.15^a^	4.28 ± 0.40^b^	6.63 ± 0.24^c^
Ash (g %)	1.58 ± 0.05^a^	1.79 ± 0.22^a^	1.76 ± 0.14^a^	1.81 ± 0.08^a^	2.22 ± 0.19^b^
Dietary fibers					
Insoluble (g %)	8.41 ± 0.12^a^	8.71 ± 0.09^a^	10.67 ± 0.25^b^	12.43 ± 0.38^c^	9.89 ± 0.16^d^
Soluble (g %)	0.85 ± 0.001^a^	1.05 ± 0.010^b^	0.97 ± 0.002^c^	0.99 ± 0.001^d^	0.86 ± 0.001^a^
Total (g %)	9.26 ± 0.15^a^	9.76 ± 0.17^a^	11.64 ± 0.20^b^	13.42 ± 0.25^c^	10.75 ± 0.21^d^
†Carbohydrate (g %)	63.61	60.04	61.01	60.88	56.40
Energy value (kcal)	378.85 ± 3.26^a^	382.32 ± 6.90^a^	382.75 ± 7.55^a^	381.10 ± 2.82^a^	392.07 ± 2.89^a^
Amylose					
Insoluble (%)	14.75 ± 0.92^a^	12.97 ± 0.67^a^	12.41 ± 0.40^a^	17.05 ± 1.53^a^	10.80 ± 0.61^b^
Soluble (%)	13.65 ± 0.14^a^	13.88 ± 0.40^a^	12.01 ± 0.35^b^	7.47 ± 0.30^c^	10.95 ± 0.13^d^
Total (%)	28.33 ± 0.41^a^	27.42 ± 0.83^a^	24.90 ± 0.17^b^	24.55 ± 0.56^c^	21.70 ± 0.73^d^
Phosphorus (mg %)	347.13 ± 7.01^a^	376.18 ± 6.14^b^	350.08 ± 6.40^a^	402.51 ± 4.88^c^	459.54 ± 8.30^d^
Oryzanol (mg %)	348.22 ± 8.40^a^	327.90 ± 5.56^a^	373.53 ± 9.04^a^	471.51 ± 4.98^b^	380.76 ± 6.11^c^

Values are mean ± standard deviation of three independent determinations. Values in the same row (in each parameter) with different superscripts (a, b, c, d, e) are significantly different at *p* < 0.05 when raw compared with the respective process. *Protein (*N* × 6.25), †Carbohydrate (By difference). %, percentage; g, gram; kscg, kilogram-force per square centimetre gauge; mg, milligram.

Germination of millet increased the crude fat content by 5.0% which is statistically significant, while roast processing significantly increases the crude fat content by 25.3% compared to raw flour. In contrast, both the steam processed flour showed a reduction (by 1.89 and 19.09%) in crude fat content compared to raw flour.

Ash content of processed millet ranged from 1.47 to 2.22%. All the processing methods increased the crude ash content compared to the raw millet, whereas roast processing method alone significantly increased the crude ash content by 40.5% compared to raw millet.

Dietary fibre (soluble, insoluble, and total) showed a slight increase in germinated millet (not significantly). Whereas heat processing methods significantly increased the dietary fibers as observed in steam processed (0 kscg: 25.7%, 1.5 kscg: 44.9%) and roasted (16.1%) millet flour samples.

The carbohydrate content by difference method fell within a range of 56.4–63.6% and where all processing methods showed reductions in carbohydrate content compared to raw millet flour.

Energy value ranged from 378.8–392.1 kcal. All the processing methods increased (Not significant) the energy value, with roast processing method sample recorded the highest value (3.49%) compared to the raw millet.

Amylose content decreased significantly in all processing methods, with roasting of millet showed the highest reduction (23.4%), followed by high-pressure (1.5 kscg) steaming (13.3%), mild 0 kscg steaming (12.1%), and germination (3.2%) treated millet compared to the raw millet.

All the processing methods increased the total phosphorus content. Germination of millet grains increased the total phosphorus content by 8.3% compared to raw. Steam processing treatment at 1.5 kscg (15.95%) and roasting (32.38%) increased the phosphorus content significantly (*p > 0.05*) compared to raw millet flour.

High-pressure steaming (1.5 kscg) processed millet had significant increase in the Oryzanol content (35.4%) which is followed by roast processing (9.3%), whereas 0 kscg steam processed (7.3%), and germinated (5.8%) of millet flour showed increase in the Oryzanol content but not at significant level compared to raw millet.

### 3.3 Functional properties

Functional Properties of raw and processed millet flour are presented in Table 3. Colour analysis of millet flour samples revealed that germinated millet flour exhibited 9.6% higher lightness (L*) than raw and heat-processed millet flours, which was accompanied by a 31.3% reduction in Δ*E* value. Redness (a*) and yellowness (b*) decreased by 1.9-fold and 14.0%, respectively, in germinated flour compared to raw. The highest b* values were observed in steamed (25.2% for 1.5 kscg sample) and roasted (24.6%) flours, which is followed by raw, with germinated flour showing the lowest yellowness.

**Table 3:** Effect of processing on functional properties of little millet.

Parameters	Raw	Germinated	Steamed (0 kscg)	Steamed (1.5 kscg)	Roasted
Colour					
L*	72.40 ± 1.64^a^	79.35 ± 1.01^b^	71.49 ± 0.95^a^	68.76 ± 1.59^a^	69.31 ± 1.40^a^
a*	0.34 ± 0.005^a^	−0.30 ± 0.002^b^	0.14 ± 0.010^c^	0.24 ± 0.001^d^	0.25 ± 0.005^e^
b*	11.37 ± 1.23^a^	9.78 ± 0.98^a^	12.56 ± 0.65^a^	14.24 ± 0.85^a^	14.17 ± 1.03^a^
∆*E*	21.26 ± 1.42^a^	14.61 ± 1.00^b^	22.63 ± 0.50^a^	25.84 ± 1.63^c^	25.34 ± 1.05^a^
Gel consistency (cm)	6.94 ± 0.23^a^	5.57 ± 0.65^a^	8.79 ± 0.10^a^	10.15 ± 1.19^b^	7.50 ± 0.26^a^
Sediment volume (mL)	4.84 ± 0.15^a^	6.06 ± 0.18^b^	5.03 ± 0.17^a^	6.67 ± 0.21^c^	10.55 ± 0.20^d^
Pasting profile					
Gelatinization temperature (°C)	82.03 ± 0.55^a^	80.40 ± 0.39^a^	84.55 ± 0.68^b^	50.25 ± 0.50^c^	50.25 ± 0.42^d^
Peak viscosity (BU)	152.45 ± 2.64^a^	124.00 ± 1.43^b^	40.58 ± 1.50^c^	12.35 ± 0.60^d^	14.30 ± 0.95^e^
Hot paste viscosity (BU)	280.60 ± 0.80^a^	273.79 ± 0.74^b^	64.03 ± 0.50^c^	14.57 ± 0.70^d^	16.15 ± 0.71^e^
Cold paste viscosity (BU)	295.06 ± 0.60^a^	289.20 ± 0.93^b^	63.52 ± 0.69^c^	14.05 ± 0.20^d^	15.05 ± 0.35^e^
Breakdown viscosity (BU)	4.33 ± 0.52^a^	9.05 ± 0.23^b^	0.19 ± 0.05^c^	1.20 ± 0.04^d^	2.05 ± 0.04^e^
Setback viscosity (BU)	147.70 ± 0.70^a^	174.08 ± 0.60^b^	23.41 ± 0.75^c^	3.34 ± 0.09^d^	4.17 ± 0.05^e^
*In-vitro* carbohydrate digestibility (%)					
Time intervals: 30 min	73.07 ± 0.42^a^	77.81 ± 0.88^b^	67.86 ± 0.53^c^	66.42 ± 0.61^d^	63.58 ± 0.55^e^
60 min	73.64 ± 0.75^a^	78.84 ± 0.90^b^	68.56 ± 0.35^c^	66.89 ± 0.45^d^	64.02 ± 0.90^e^
120 min	75.40 ± 0.91^a^	79.98 ± 0.55^b^	69.43 ± 0.50^c^	67.86 ± 0.92^d^	64.81 ± 0.65^e^

Values are mean ± standard deviation of three independent determinations. Values in the same row (in each parameter) with different superscripts (a, b, c, d, e) are significantly different at *p* < 0.05 when raw compared with the respective process. %, percentage; a*, redness; b*, yellowness; BU, Brabender Units; cm, centimetre; Δ*E*, total colour difference; kscg, kilogram-force per square centimetre gauge; L*, lightness; mL, millilitre.

The gel consistency of millet flour increased after steaming at 0 kscg (26.5%) and roasting (8.0%) treatment but not at significant level compared to the raw millet. Steaming at 1.5 kscg significantly increased the gel consistency by 46.2%, whereas germinated millet flour exhibited a 19.4% reduction compared to raw millet flour.

Sediment volume in millet flour ranged from 4.8 to 10.5 mL across all the treated millet flour. The maximum significant increase (118.0%) occurred in roasted millet flour. Whereas upon germination (25.2%) and steaming at 1.5 kscg (37.8%) significantly increased sediment volume compared to raw millet flour.

**Figure 2:**
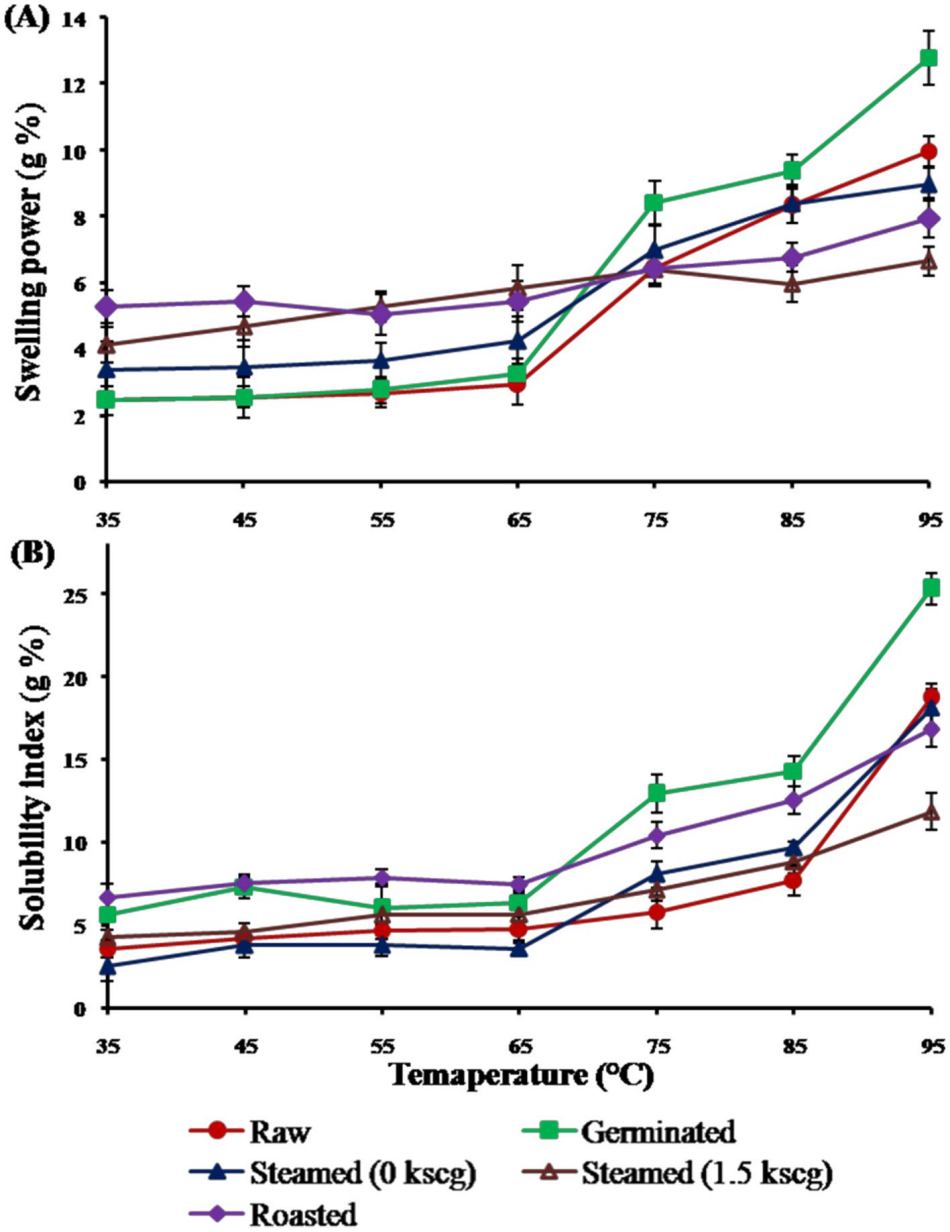
**(A)** Swelling and **(B)** solubility pattern of raw and processed little millet flour. Values are mean ± standard deviation of three independent determinations. kscg, kilogram-force per square centimetre gauge.

Both raw and germinated millet exhibited lower swelling power (<4 g%) in the temperature range of 35–65 °C. Beyond this range, swelling power increased rapidly, with germinated millet flour showing the highest values (>12 g%), followed by raw, 0 kscg, roasted, and 1.5 kscg steam-processed millet flour, as illustrated in Figure 2A. In contrast, steamed and roasted millet flour exhibited higher swelling power up to 75 °C, followed by a slight decline at temperatures above this point.

Solubility percentage increased consistently with increase in temperature in all the millet flours (Figure 2B). Germinated millet flour showed the highest increase (25 g%), following a pattern similar to swelling power. Whereas both steam-processed and roasted millet flours exhibited higher solubility at lower temperatures (35–85 °C) compared to raw millet, a subsequent decrease in solubility was observed in all heat processed flours at 85 °C temperature.

**Figure 3:**
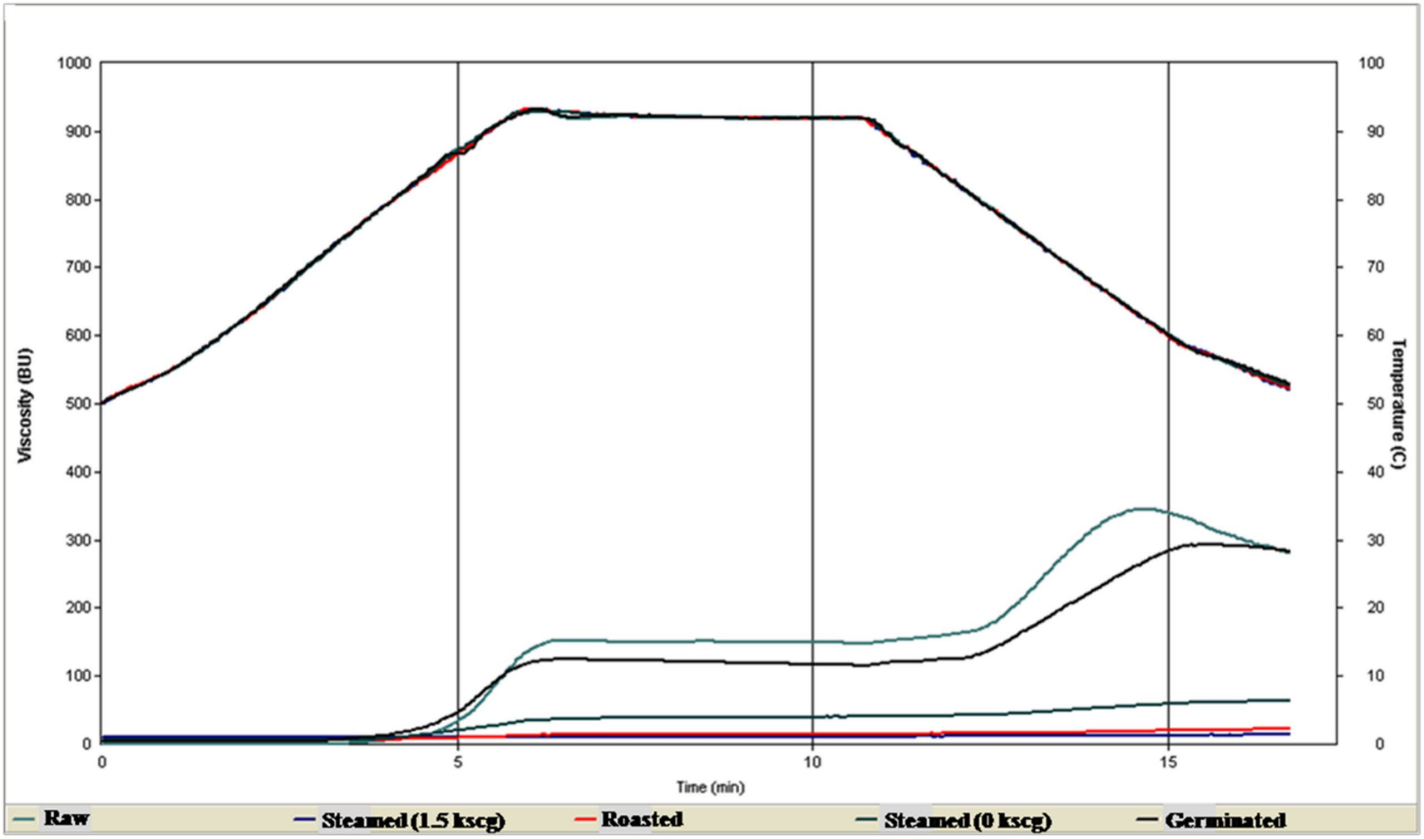
Pasting profiles of the raw and processed little millet flour. kscg, kilogram-force per square centimetre gauge.

The pasting profile of raw and processed millets are presented in Table 3 and Figure 3. Both steam (1.5 kscg) processed and roasted millet flour exhibited a significant (*p* < 0.05) reduction in gelatinization temperature (GT) compared to raw millet (82.03 °C), with roasted flour exhibiting the lowest GT (50.2 °C). In contrast, 0 kscg steam processing and germination showed only slight variations in GT relative compared to raw millet flour, which is not at statistically significant level.

The peak viscosity was highest in raw flour (152.45 BU) and lowest in roasted and 1.5 kscg steam-processed flour. Hot paste viscosity and cold paste viscosity also followed this same trend, being higher in raw flour and reduced upon heat processing methods. Germinated flour showed lower peak viscosity (124.00 BU), hot paste viscosity (273.79 BU), and cold paste viscosity (289.20 BU) compared to raw flour (152.45, 280.60, and 295.06 BU, respectively); due to degradation of the starch by the enzyme activity.

The breakdown viscosity (BD) and setback viscosity (SB) decreased significantly (*p* < 0.05) in all heat-treated millet flours (0 kscg and 1.5 kscg steamed, and roasted millet flours). Conversely, germinated millet flour showed increased BD (90.5 BU) and SB (174.08 BU) compared to raw millet flour (4.33 and 147.70 BU, respectively).

The *in vitro* carbohydrate digestibility of raw and processed millet flour increased progressively with digestion time at 30, 60, and 120 min (Table 3). Germinated millet flour exhibited significantly higher digestibility at all time intervals (77.81, 78.84, and 79.98%, respectively) compared to raw millet flour (73.07, 73.64, and 75.40%). In contrast, steam processing (both 0 kscg and 1.5 kscg) and roasting significantly reduced digestibility across all time points. Among the processed samples, roasted millet flour showed the lowest digestibility (63.58, 64.02, and 64.81% at 30, 60, and 120 min, respectively), followed by steaming (1.5 kscg) and steaming (0 kscg) millet flours. These results indicate that germination enhances carbohydrate digestibility, whereas heat processing method, particularly roasting, reduces it significantly (*p* < 0.05).

## 4 Discussion

Conventional processing techniques such as germination, steaming, and roasting play a critical role in altering the structural and physicochemical properties of any millets for diverse functional applications. The moisture content of little millet increased significantly after germination, steaming, and roasting methods. This increase is mostly due to their inherent ability to absorb and retain water during the processing. The higher moisture content observed under high steam pressure can be attributed to endosperm cooking and subsequent starch retrogradation, which in turn enhances water-binding capacity within the grains. Similarly, roasting increased moisture content is due to starch gelatinization, as observed in paddy (18) and millets processing studies (21). Increase in moisture content during germination process, is explained by structural modifications such as increased porosity and water-holding capacity and also proven in many other studies (22–24).

Moreover, Equilibrium moisture content upon soaking (EMC-S) increased significantly with germination, steaming, and roast treatment methods. These thermal and biological treatments alter the internal matrix loosening of starch granules, development of capillary pores, and increased surface area, which enhance moisture retention. Additionally, vapor pressure differences between grain and environment facilitate moisture migration into kernels. Similar increases in EMC-S have also been reported in millet processing studies by several authors (25, 26).

Importantly, unaltered geometric mean diameter and sphericity index indicates that grain overall shape and external structure stayed intact after the conventional processing methods. This observation indicates that the applied treatments predominantly act on the internal architecture of the kernel rather than its external morphology. However, true density and grain weight decreased, which can be mechanistically explained by internal structural loosening, increased moisture absorption and retention, and compositional transformation. Similar observations have been reported by many authors (21, 26, 27) in proso, barnyard and foxtail millets, where germination reduced density of the grains without affecting grain dimensions.

There was significantly decrease in bulk density upon germination and heat processing methods. This reduction is mainly due to the formation of void spaces and air pockets between the husk and caryopsis, which lowers mass per unit volume (28). Correspondingly, porosity increased, especially under high-pressure steaming, due to structural expansion and cellular breakdown. Similar trends have been reported in other millets and grains, including processed cashew nuts by other authors (21, 26, 29).

Hardness decreased significantly after germination, while it increased in steaming and roast processing method. This reduction in hardness during germination is due to enzymatic degradation of cell wall components (by xylanases, & proteases etc) and partial starch hydrolysis by amylases, resulting in softer grains (22, 30). In contrast, the increase in hardness after heat treatment is due to the formation of protein–starch matrices, which restrict granule movement and increase rigidity as observed by other authors in little millet and other cereal grains (31, 32).

SEM microscopic observations confirmed structural changes in starch architecture during processing methods. Germination of millet resulted in granule swelling and loosening due to hydration and enzymatic activities, whereas steam treatment promoted gelatinization with swollen and aggregated structures, and roasting led to complete gelatinization followed by amylose retrogradation as reflected in the SEM images. These microstructural changes explain the observed differences in texture and physical properties after processing methods by other authors (33, 34).

Porosity of the little millet grains increased significantly after all 3 processed methods with the highest values observed in 1.5 kscg stream processed millet grains. This increase is due to swelling and partial breakdown of cellular structures, leading to formation of internal void spaces. Germination of grains promotes enzymatic softening and expansion, while steaming of grains induces the starch gelatinization and protein denaturation. In contrast to this, roasting may stabilize or slightly reduce porosity due to matrix shrinkage caused by dehydration as observed (32) in other cereal grains.

Protein content increased significantly after all the three processing methods in little millet. This increase is attributed to protein synthesis during germination and concentration effects resulting from carbohydrate loss during heat treatments such as steaming and roast processing. Additionally, reduction of anti-nutritional factors improves protein availability as observed by other authors (26, 30, 35).

Fat content of little millet grains increased following roasting and germination process, whereas it decreased in steam processed millets. This increase observed during roasting may be attributed to the breakdown of protein–lipid and carbohydrate–lipid complexes, which enhances lipid extractability (21). Similarly, several studies have reported that germination leads to an increase in fat content due to lipid mobilization and structural modifications within the cell matrix, including degradation of cell wall components and structural lipids, as well as the release or synthesis of new lipophilic compounds (36, 37). In contrast, steaming results in a reduction of fat content, possibly due to the formation of amylose–lipid complexes that limit lipid extractability, as reported in studies conducted on proso millet (33, 35, 38).

Ash content increased significantly across all processing methods. This increase can be primarily attributed to the concentration of minerals resulting from dry matter loss during germination and heat processing. Similar trends have been reported in various millet varieties, where processing led to an apparent increase in ash content due to the reduction of other compositional components (21, 39, 40).

Dietary fibre content increased following processing, which might be due to the disruption of cellular structures and modifications in cell wall polysaccharides (41). Germination might enhance soluble fibre content through enzymatic activity that partially degrades complex carbohydrates (42). In contrast, heat- processing treatments such as steaming and roasting, might increase the relative proportion of insoluble fibre, possibly due to the concentration effect and structural rearrangement of cell wall components (43). Similar trends have been reported in foxtail millet and other millets in recent studies (26, 30, 44–46).

Carbohydrate content decreased significantly during germination and heat processing. This reduction during germination might be due to the activity of *β*-amylase, which hydrolyzes starch into simpler sugars that are subsequently utilized by the growing embryo (40, 47). Similar reductions have been reported in pearl millet and other cereal grains (45). Additional losses during steaming and roasting might be due to the leaching of soluble sugars and the occurrence of Maillard reactions (33).

Energy values increased after processing, particularly following roasting. This increase might be due to the higher concentrations of protein and fat resulting from the reduction of moisture and other components during processing. Similar trends have been reported in pearl millet during sprouting and roasting (45).

Amylose content decreased significantly after processing. This reduction might be due to enzymatic degradation during germination, as well as thermal disruption of starch structure during heat treatments process, which in turn affects its functional properties (48).

Phosphorus content increased significantly with processing, particularly during roasting and steaming. This increase might be due to the combined effects of nutrient concentration and enzymatic activity during germination, along with the breakdown of anti-nutritional factors such as phytates, which enhances mineral bioavailability. Similar findings have been reported in little millet and other millet grains by many authors (26, 45, 49).

The highest oryzanol content was observed after high-pressure steaming. This increase might be due to the hydrothermal release of bound compounds, which enhances extractability, while the removal of moisture and structural changes such as surface collapse may further facilitate the release of bound oryzanol in cereal grains, particularly in rice bran (50, 51).

Colour parameters showed increased lightness after germination, whereas steaming and roasting resulted in darker coloured flours. The increase in lightness might be due to biochemical changes during germination, including the degradation of pigments and structural modifications. In contrast, the darkening of flour observed after heat treatments might be attributed to non-enzymatic browning reactions, such as Maillard reactions, along with pigment degradation (21, 40).

Gel consistency increased after steaming and roasting but decreased following germination. The increase might be due to the formation of pre-gelatinized starch during heat treatments, which enhances water absorption and gel formation (52). In contrast, the decrease during germination might be attributed to enzymatic degradation of starch and the leaching of soluble components, leading to weaker gel structure (53, 54).

Sediment volume increased after roasting and germination. This increase might be due to starch pre-gelatinization during roasting and enzymatic modifications during germination, both of which enhance water absorption and swelling capacity (53). Swelling power and solubility increased following the processing treatments. The post-germination upsurge in swelling power might be due to the enzymatic degradation of the starch matrix, which enhances hydration properties in the millet. On a contrary, steam and roast-treated grains exhibited pre-gelatinization effects that facilitates hydration at lower temperatures but may restricts swelling at higher temperatures due to structural constraints (26, 55). Moreover, germination significantly increases solubility, which might be driven by partial starch hydrolysis and subsequent release of low-molecular-weight compounds. Similarly, heat-treated millets exhibited increased solubility, probably due to the presence of pre-gelatinized starch networks as reported in other studies (27, 55, 56). Gelatinization temperature slightly increased after steaming and roasting, which might be due to the rearrangement of starch into more ordered and compact crystalline regions, along with the formation of cross-linkages that require higher energy for disruption (57–59). In contrast, peak, breakdown, and final viscosities decreased following heat processing, which might be attributed to protein denaturation, strengthened starch–protein interactions, and partial disintegration and reorganization of starch granules, all of which restrict swelling and reduce water-binding capacity (60, 61). Such reductions in viscosity parameters after thermal treatments have been widely reported in cereal grains, indicating limited paste development and altered granular integrity. The observed decrease in breakdown viscosity after heat treatment further suggests improved resistance to thermal treatment, reflecting enhanced paste stability (62). Notably, germination decreases viscosity by enzymatic degradation of starch and amylolytic activity, which reduces its molecular weight and disturbs the granule architecture (63, 64). At the same time, germinated little millet exhibited higher breakdown viscosity, which might be due to weakened granule integrity and increased susceptibility to disintegration under heating and shear condition, this is in consistent with previous findings that germination significantly alters starch structure and pasting behavior (22, 58, 65).

Carbohydrate digestibility increased significantly after germination, which might be due to enzymatic hydrolysis and enhanced accessibility of starch resulting from the breakdown of complex macromolecules. This process typically increases the proportion of rapidly digestible starch (RDS), while reducing resistant starch (RS), thereby potentially elevating the glycaemic response (66). In contrast, steaming and roasting reduced digestibility, particularly after roasting, which might be attributed to the formation of resistant starch through amylose retrogradation, as well as the development of Maillard reaction products that hinder enzymatic access. These changes are often associated with an increase in slowly digestible starch and resistant starch fractions, contributing to a lower predicted glycaemic response. Similar findings have been reported in foxtail millet and other pulses (46, 67).

## 5 Conclusion

This study demonstrates that conventional processing methods such as germination, steaming, and roasting significantly modify the physical properties, nutritional composition, and functional characteristics of little millet, thereby enabling diverse opportunities for various product development. Germination resulted in softer grains with improved digestibility and minimal colour change, making it suitable for applications such as infant foods and quick-cooking products. In contrast, Roasting and high-pressure steaming Treatment technique, improved grain hardness and elevated protein, dietary fibre and oryzanol content, while reduced *in vitro* carbohydrate digestibility and indicating possible low-glycaemic uses. These properties are the foundation of their prospective use in low-glycaemic, high-satiety food compositions important to control the metabolic health management. Importantly, these simple and scalable processing approaches enhance the value of little millet without the need for advanced technologies. Evaluation of functional parameters, including pasting properties, swelling power, and in-vitro digestibility provide critical insights which will help this grain for food processing applications, while the observed nutritional improvements highlight its potential advantage over traditionally available cereals. However, the results are restricted to *in vitro* tests and their physiological significance has to be validated by human intervention trials. Sensory assessment and customer acceptance studies are also required to help product development. The current research was limited to a single variety (CO-Samai-4) and more exploration across other kinds are necessary. Overall, the findings suggest the potential of the processed tiny millet as a functional ingredient for developing nutritionally improved food products. We aim for our future research focusing on validating the postprandial glycaemic response, sensory acceptability, and nutrient bioavailability of multiple little millet varieties as well as their products, along with pilot-scale validation, to facilitate translation from laboratory findings to commercial applications.

## Data Availability

The original contributions presented in the study are included in the article/supplementary material, further inquiries can be directed to the corresponding authors.
